# New Pseudomonas Bacterial Strains: Biological Activity and Characteristic Properties of Metabolites

**DOI:** 10.3390/microorganisms11081943

**Published:** 2023-07-29

**Authors:** Tatiana M. Sidorova, Natalia S. Tomashevich, Valeria V. Allahverdyan, Boris S. Tupertsev, Yuri I. Kostyukevich, Anzhela M. Asaturova

**Affiliations:** 1 Federal Research Center of Biological Plant Protection, 350039 Krasnodar, Russia; 0166505@mail.ru (T.M.S.); lera_arm@mail.ru (V.V.A.);; 2 Phystech School of Biological and Medical Physics (FBMF), Moscow Institute of Physics and Technology, 141701 Dolgoprudny, Russia; 3 Center of Molecular and Cellular Biology (CMCB), Skolkovo Institute of Science and Technology, 121205 Moscow, Russia

**Keywords:** *Pseudomonas*, metabolites, *Fusarium*, antagonistic activity

## Abstract

This paper investigates the antagonistic and plant growth promotion activity of the new indigenous bacteria antagonist strains *P. chlororaphis* BZR 245-F and *Pseudomonas* sp. BZR 523-2. It was found that on the 10th day of cultivation, BZR 245-F and BZR 523-2 exhibit an antagonistic activity against *F. graminearum* at the level of 59.6% and 15.1% and against *F. oxysporum* var. *orthoceras* at the level of 50.2% and 8.9%, respectively. Furthermore, the BZR 523-2 strain stimulated the growth of winter wheat seedlings more actively than the BZR 245-F strain. When processing seeds of winter wheat, *Pseudomonas* sp. BZR 523-2 indicators were higher than in the control: plant height increased by 10.3%, and root length increased by 18.6%. The complex characteristic properties of the metabolite were studied by bioautography and HPLC-MS. Bioautography proved the antifungal activity of phenazine nature compounds synthesized by the new bacterial strains. We qualitatively and quantitatively analyzed them by HPLC-MS analysis of the strain sample metabolites. In the BZR 245-F sample, we found more phenazine compounds of various types. Their total phenazine concentration in the BZR 245-F was more than five times greater than in the BZR 523-2. We defined crucial differences in the quantitative content of the other metabolites. Despite the difference between new indigenous bacteria antagonist strains, they can be used as producers of effective biopesticides for sustainable agriculture management.

## 1. Introduction

Rhizobacteria that stimulate plant growth are an alternative to chemical crop protection in agriculture. For decades, mineral fertilizers and pesticides used in agriculture have degraded soil and plant health, increasing the constant risk of plant pathogens. Bacteria of the genus *Pseudomonas* are considered promising agents among plant growth-promoting rhizobacterium. They can colonize the root system, produce exopolysaccharides, siderophores, phytohormones, solubilize phosphorus, potassium, and zinc, and form biofilms.

Due to the developed mobility apparatus and a wide range of substrates used for their vital activity, covering all the main compounds of plant root exudates, *Pseudomonas* strains are ideal biocontrol agents for inhibiting crop diseases [1]. Many *Pseudomonas* are capable of producing antimicrobial compounds, like phenazines, pyrrolnitrin, phenazine-1-carboxamide, phenazine-1-carboxylic acid, 2,4-diacetylphloroglucinol, rhamnolipids, pyoluteorin, oomycin A, ecomycins, viscosinamide, cepacyamide A, pyocyanin, butyrolactones, N-butylbenzenesulfonamide, pseudomonic acid, azomycin, cepafungins, and caralicin [2,3]. A number of biocidal metabolites produced by *P. chlororaphis* O6 include gaseous compounds, such as hydrogen cyanide, butanediol, and hydrocarbons that can move through air channels in the soil [4,5]. In addition, *Pseudomonas* produce chitinases, glucanases, and proteases. Their production is mainly induced by the biomass of fungal pathogens and their cell walls [6].

The involvement of secondary metabolites in biological control was first observed through the correlation of pathogen inhibition in vitro and disease suppression in vivo [2]. The potential of *Pseudomonas* strains producing phenazines and their derivatives has been extensively studied, since these metabolites have fungicidal properties [6]. Isolated natural phenazines showed an antifungal activity at or above the level of commercial fungicides. The occurrence and concentration of these substances strongly depended on the strain and affected the biological effectiveness of microorganisms. The highest capacity for biocontrol was noted in *P. chlororaphis* and *P. fluorescens*. Thus, *P. fluorescens* 2–79, which produces phenazine-1-carboxylic acid, *P. aureofaciens* 30–84, which produces 2-hydroxyphenazine-1-carboxylic acid and 2-hydroxyphenazine, and *P. chlororaphis* PCL1391, which produces pyocyanin, suppress *Gaeumannomyces graminis* var. *tritici* [7,8,9].

In recent decades, many studies have revealed the potential of natural lipopeptide compounds for agricultural use. They are surface-active compounds produced by a wide range of microorganisms. Despite their chemical diversity, they are all amphiphilic molecules consisting of hydrophobic and hydrophilic fragments. The cyclic lipopeptides produced by *Pseudomonas* are divided into four main groups: viscosins, amphysins, tolaazines, and syringomycins. The viscosin group consists of lipopeptides with nine amino acids, whereas the substances of the amphysin group contain 11 amino acids in the peptide portion. For both groups, the lipid tail in most cases is 3-hydroxydecanoic acid. The lipopeptides in the tolaazine group are more diverse due to the differences in the length of the peptide chain (19–25 amino acids) and a lipid tail (3-hydroxydecanoic acid or 3-hydroxyoctanoic acid). They contain several unusual amino acids, such as 2,3-dehydro-2-aminobutyric acid and homoserine. Lipopeptides of the syringomycin group contain amino acids, such as 2,4-diaminobutyric acid, and a C-terminal residue of chlorinated threonine. Masitolide A from the viscosin group, produced by the *P. fluorescens* SS101 strain, exhibits a direct antagonism against *P. infestans* and induces disease resistance in tomato crops [8,9,10,11].

Their use in agriculture is aimed at bioremediation, crop growth regulation, and crop protection [4,12]. The widely varying biocidal (bactericidal, fungicidal, insecticidal) activity of lipopeptides is of great interest to researchers. These properties are associated with their ability to disrupt the permeability of the bacterial cytoplasmic membrane, form pores in it, and destroy it. Taxonomic selectivity and pore formation mechanisms in the plasmalemma are different for lipopeptides of different families, which causes their specific biological activity. Plant-associated microorganisms (*Pseudomonas* sp. and *Bacillus* sp.) induce the host phyto-immune system by triggering the jasmonic–ethylene signaling pathway mediated by the NRP-1 signaling protein, with the participation of bacterial determinants, including lipopeptides [12].

The other most intensively studied *Pseudomonas*-synthesized class of surface-active glycolipids is rhamnolipids. These glycolipids are synthesized as a mixture of compounds with one or two rhamnose residues forming a polar hydrophilic head linked by a beta-glycosyl bond to one or two 3-hydroxy fatty acids in the form of hydrophobic tails. Rhamnolipids are considered to be readily biodegradable and have low toxicity. The properties of rhamnolipids allow them to be used in a variety of fields, including medicine, cosmetics, food, oil, and agriculture. Natural rhamnolipids are known to have antimicrobial properties and the ability to induce plant resistance mechanisms. For example, they have proved to inhibit the growth of various phytopathogenic fungi, such as *Botrytis cinerea*, *Fusarium solani*, *Phytophtora* sp. Hence, rhamnolipids are believed to be promising compounds for plant protection [5].

The use of metabolites for crop protection is of increasing interest, since they are generally biodegradable and environmentally friendly. Therefore, we consider the bacteria that produce them as potential agrobiotechnology objects—the basis for the development of biological crop protection products. Over the past two decades, the synthesis of antibiotics using rhizospheric bacteria has been one of the most successful and well-studied methods of biological control of various plant diseases [9].

The objective of this work is to study the biological activity and characteristic properties of metabolites of the new bacteria antagonists *P. chlororaphis* BZR 245-F and *Pseudomonas* sp. BZR 523-2, which we consider promising for the development of biopreparations for plant protection.

## 2. Materials and Methods

### 2.1. Bacterial and Fungal Strains

In this research, we used two indigenous strains of *Pseudomonas* (*P. chlororaphis* BZR 245-F and *Pseudomonas* sp. BZR 523-2) isolated from the rhizosphere of winter wheat in the Kuban Region (Krasnodar 45.035470 N, 38.975313 E and Gulkevichsky District 45.318371 N 40.682679 E, respectively). They were identified by 16-S RNA and were included in the Bioresource Collection (BRC) of the Federal State Budgetary Scientific Institution “Federal Research Center of Biological Plant Protection” (FSBSI FRCBPP) “The State Collection of Entomoacariphages and Microorganisms” as a promising bacterium. It has been previously shown that the *P. chlororaphis* BZR 245-F and *Pseudomonas* sp. BZR 523-2 strains have lipolytic activity, while the *Pseudomonas* sp. BZR 523-2 strain also has a high chitinolytic activity [13]. Both strains are not phytotoxic for winter wheat seedlings with cut root systems [13]. *P. chlororaphis* BZR 245-F isolated from the wheat rhizosphere was found to inhibit the development of downy mildew and Septoria blight of soybean [14,15], and contributes to the suppression of Septoria blight and late blight of tomato [16]. *Pseudomonas* sp. BZR 523-2 has a plant growth promotion effect [17]. It is crucial to characterize *Pseudomonas* strains and understand their mechanisms of effective plant interaction to assess their biocontrol potential.

As a test object to determine the ability of bacteria to exhibit fungicidal properties, we used the strains *F. graminearum* Schwabe BZR F-4 and *F. oxysporum* var. *orthoceras* App. et Wr. BZR F-6 from the BRC of the FSBSI FRCBPP—*Fusarium* pathogens of various crops.

### 2.2. Chemicals and Reagents

We used the King B medium (peptone 20 g, glycerin 15 g, MgSO_4_·7H_2_O 1.5 g, K_2_HPO_4_·3H_2_O 1.97 g, distilled water 1 L) to grow the bacterial strains *P. chlororaphis* BZR 245-F and *Pseudomonas* sp. BZR 523-2.

We used potato-glucose agar (potatoes 500 g, glucose 20 g, agar 20 g, water 1 L) to grow a pure culture of *F. graminearum* Schwabe BZR F-4 and *F. oxysporum* var. *orthoceras* App. et Wr. BZR F-6 and for the dual culture plate assay.

We used potato-glucose medium (potatoes 500 g, glucose 20 g, water 1 L) for the bioautography method.

We used the standard metabolite phenazine (98% purity, Merck KGaA, Darmstadt, Germany) to evaluate the antifungal activity of bacterial metabolites by bioautography and to analyze metabolites qualitatively and quantitatively for HPLC-MS.

The solvent for thin layer chromatography (TLC) was ethyl acetate/ethanol/water (40:15:15).

The HPLC solvents, i.e., water (H_2_O), acetonitrile (ACN), and formic acid (HCOOH), were obtained from Merck KGaA (Darmstadt, Germany).

### 2.3. Growth Conditions

The liquid culture of the bacteria *P. chlororaphis* BZR 245-F and *Pseudomonas* sp. BZR 523-2 was obtained by batch cultivation on an Excella E25 Incubator Shaker (New Brunswick Scientific, Enfield, CT, USA) for 36 h at 26 °C under the King B medium. We used a method of successive dilutions to analyze the quantitative growth patterns of the studied strains. The experiment was run in triplicate [18]. The grown colonies were counted using the QCount Colony Counting System (Spiral Biotech, Norwood, MA, USA).

Pure cultures of the fungal strains *F. graminearum* Schwabe BZR F-4 and *F. oxysporum* var. *orthoceras* App. et Wr. BZR F-6 were grown in a potato-glucose agar for seven days at 24 °C.

### 2.4. Antagonistic and Plant Growth Promotion Activity of Indigenous Pseudomonas Strains

The antagonistic activity of bacterial strains was determined by dual culture plate assay in a potato-glucose agar [18]. A mycelial plug of *F. graminearum* BZR F-4 and *F. oxysporum* var. *orthoceras* BZR F-6 was placed in a Petri dish and a bacterial strain was plated at the distance of 6 cm from fungus. Pure cultures of the fungal pathogen and bacteria placed separately were used as positive and negative controls. Dual cultures were incubated for 10 days at 28 °C. The growth of the fungal colonies was measured every five days. The presence of a sterile zone and its size, as well as the fungal color, density, and direction of mycelial growth, were registered. Antagonistic activity was calculated according to the following formula: % inhibition = [1 − (Fungal growth/Control growth)] × 100 [19].

The growth stimulating activity of the bacterial strains *P. chlororaphis* BZR 245-F and *Pseudomonas* sp. BZR 523-2 was determined by a pot experiment. Unsterilized seeds of winter wheat (Batko variety) were soaked for two hours in two-day bacterial cultures 10^9^ CFU/mL obtained by washing bacteria from Petri dishes and adding tap water to reach a volume of 50 mL. After 2 h, the seeds were removed from bacterial suspensions and dried on filter paper. After 20–24 h, 10 seeds were sown in each of the 30.45 L pots filled with sterilized sand. The pots were stored in a greenhouse at 24–28 °C and 11,000 lux. The length of the roots, height of the stems, and seedlings’ dry biomass were measured after 14 days. The root lengths and stem heights were measured for 30 replicates. Due to the small size of individual crops, the dry biomass of the crops was measured by weighing each of the three pots. The experiment was performed twice.

Data from the experiments were subjected to analysis of variance (ANOVA) and the means were compared by Duncan’s multiple range test (*p* < 0.05). All statistical analyses were performed in Microsoft Excel (standard deviation) and Statistica Version 13.5.0.17.T. (ANOVA, normality of data, and Duncan’s multiple range test).

### 2.5. Sample Preparation

**Figure 1:**
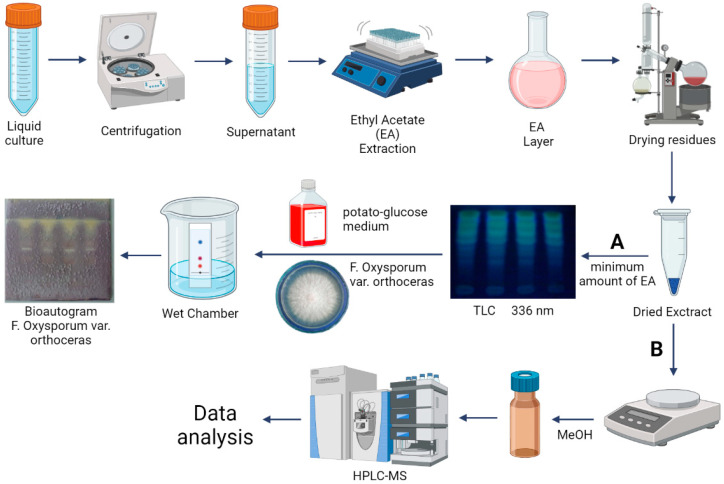
Sample preparation and analysis stages. A—Isolation, purification of bacterial exometabolites and bioautography with *F. oxysporum* var. *orthoceras*; B—HPLC-MS sample preparation.

To prepare a working solution for ascending thin layer chromatography (TLC) and high-performance liquid chromatography–mass spectrometry (HPLC-MS) analysis, the liquid culture of bacteria was centrifuged at 10,000 rpm for 30 min (Eppendorf, 5810R, Hamburg, Germany). The supernatant was mixed with ethyl acetate (1:3) and stirred on a shaker for 1 h at room temperature. The ethyl acetate extract was evaporated on an RV 10D S99 rotary vacuum evaporator (IKA, Staufen im Breisgau, Germany) until a dry residue appeared. The dry residue was washed off with a small amount of ethyl acetate (Figure 1).

### 2.6. Isolation and Purification of Bacterial Exometabolites

Isolation and purification of bacterial exometabolites were carried out by TLC. For the TLC analysis, the dry residue was washed off with a minimum amount of ethyl acetate and chromatographed on Kieselgel 60 chromatographic plates (Merck, Darmstadt, Germany), layer thickness—2 mm, and solvent—ethyl acetate/ethanol/water (40:15:15) [20,21]. We used this to study the approximate structure and properties of metabolites.

### 2.7. Evaluation of the Antifungal Activity of Bacterial Metabolites by Bioautography

We used TLC plates as a preliminary step to analyze the antifungal activity of the studied strains by the bioautography method. Antifungal metabolites were detected by bioautography with a test culture of the fungus *F. oxysporum* var. *orthoceras* BZR F-6. This fungus is a widespread causative agent of wheat common root rot. Moreover, this test culture is methodically acceptable as the mycelium growth pattern of this fungus provides a clear picture on the bioautography [20,21].

The TLC plates were sprayed with potato-glucose medium after removing traces of solvents. After that, the test fungus suspension (*F. oxysporum* var. *orthoceras* BZR F-6) was applied. Thereafter, the plates were incubated in a humid chamber for 48 h at 28 °C. Active components were identified by the formation of zones of inhibition and the decrease in the intensity of the growth of the fungus (Figure 1A).

### 2.8. HPLC-MS Conditions

HPLC-MS working solutions with a concentration of 100 μg/mL in MeOH were prepared from the dry residues of *P. chlororaphis* BZR 245-F and *Pseudomonas* sp. BZR 523-2. (Figure 1B).

All experiments were carried out on a Dionex 3000 UltiMate combined with a QExactive Orbitrap (Bremen, Germany). We used a Hypersil Gold C8 (2.1 × 50 mm, 1.8 µm) HPLC column. The mobile phase A involved 0.1% formic acid in a 5% aqueous solution of acetonitrile. The mobile phase B involved 0.1% formic acid in acetonitrile delivered at the following gradient at a flow rate of 0.600 mL/min: 0–1.0 min 2% B, 1.0–16.0 min 2.0–90.0% B, 16.0–18.0 90.0% B, 18.0–18.1 min 90.0 to 2.0% B, and 18.1–20.0 min 2.0% B. The injection volume was 1 μL. The resolving power was 35,000 (for *m*/*z* = 200). The sheath, aux, and spare gases (N_2_) were set to 45, 15, and 5, respectively. The spray voltage was 4.1 kV (for positive and negative ionization modes), and the temperature of the desolvating capillary was 320 °C. The S-Leans RF level was 50, and the source temperature was set to 200 °C.

Open screening for the metabolites was performed by full MS (100–1000 and 400–2000 *m*/*z*) followed by data-dependent analysis (DDA) in both positive and negative ionization modes. To prevent contamination of the HPLC column between the samples, blank solutions of MeOH were analyzed.

### 2.9. Quantitative Analysis of Phenazine Nature Compounds

To quantify the content of phenazine and other structurally similar secondary metabolites (pyocyanin, phenazine-1-carboxylic acid, 1-phenazinol, 2-hydroxyphenazine-1-carboxylic acid, and phenazine-1-carboxamide) solutions of phenazine (98% purity) 1–2.5–5–10–25–50–100–250–500 ng/mL were prepared and analyzed at the same HPLC-MS conditions by full MS 100–1000 *m*/*z*, followed by DDA, as mentioned above.

### 2.10. HPLC-MS Data Analysis

The spectra were processed using Xcalibur™ 4.4 software (Thermo Fisher Scientific, Houston, TX, USA). The search for the metabolites was carried out both in an automatic mode using the Compound Discoverer™ 3.2 program (Thermo Fisher Scientific, Houston, TX, USA) and manually using the PubChem and LOTUS databases. The interpretation of the mass spectra was carried out both manually and using the SkolmiX v2 open web application (https://skolmix.anvil.app/, accessed on 20 April 2023). The calibration curve for phenazine standard solutions was constructed on an Xcalibur™ Quan Browser.

## 3. Results

### 3.1. Antagonistic and Plant Growth Promotion Activity of Indigenous Pseudomonas Strains

*P. chlororaphis* BZR 245-F and *Pseudomonas* sp. BZR 523-2 were found to have an antagonistic activity against *F. graminearum* and *F. oxysporum* var. *orthoceras* (Table 1). The *Pseudomonas* sp. BZR 523-2 strain showed a lower antagonistic activity than the *P. chlororaphis* BZR 245-F strain.

Furthermore, other indicators were tested for plant growth stimulating activity (Table 2).

The *Pseudomonas* sp. BZR 523-2 strain stimulated winter wheat plants growths more actively than the *P. chlororaphis* BZR 245-F strain.

It can, therefore, be concluded that both strains are promising agents for plant growth promotion and for biocontrol.

### 3.2. Isolation of Bacterial Metabolites by TLC

**Figure 2:**
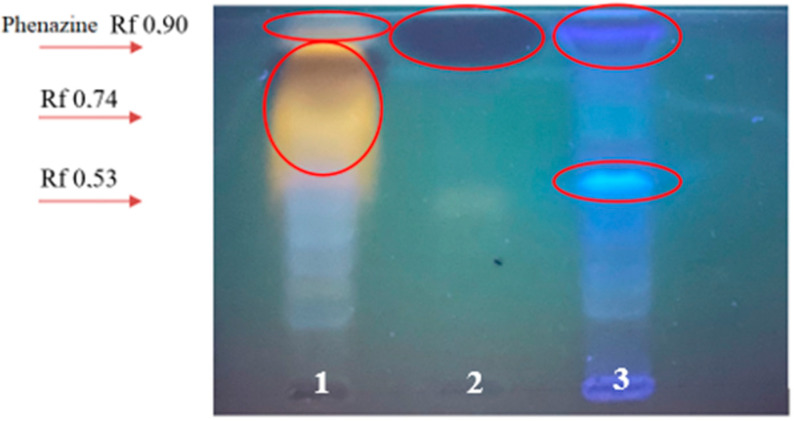
TLC (Kieselgel 60 chromatographic plates (Merck, Darmstadt, Germany), layer thickness 2 mm, solvent: ethyl acetate/ethanol/water (40:15:15)) of an ethyl acetate extract of supernatants of *Pseudomonas* bacteria strains and standard metabolite at 366 nm. 1—*P. chlororaphis* BZR 245-F; 2—phenazine standard solution; 3—*Pseudomonas* sp. BZR 523-2. Red circles indicate metabolite zones.

We observed a large number of zones glowing in different colors on a TLC plate under UV—366 nm (Figure 2). Moreover, the zones of the glowing metabolites differed in the studied *Pseudomonas* strains.

**Figure 3:**
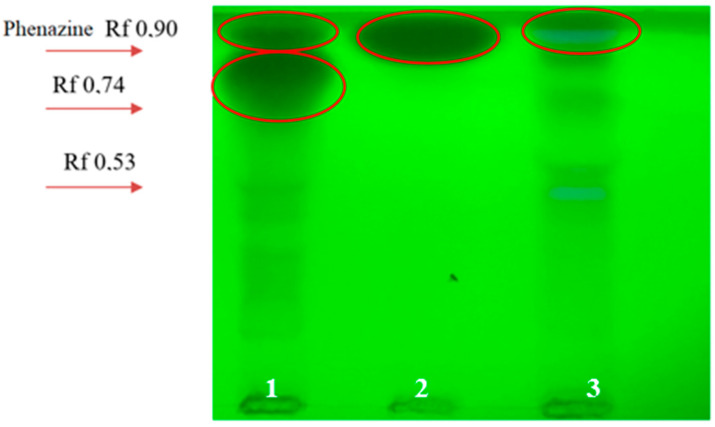
TLC (Kieselgel 60 chromatographic plates (Merck, Darmstadt, Germany), layer thickness 2 mm, solvent: ethyl acetate/ethanol/water (40:15:15)) of an ethyl acetate extract of supernatants of *Pseudomonas* bacteria strains and standard metabolite under UV—254 nm. 1—*P. chlororaphis* BZR 245-F; 2—phenazine standard solution; 3—*Pseudomonas* sp. BZR 523-2.

We found absorption zones of varying intensity on a TLC plate under UV—254 nm. It is safe to conclude that not only mono-compounds were obtained, but also complexes of metabolites. We assume phenazine metabolites as a group of compounds with the same chromatographic mobility (Rf 0.90) under given conditions (Figure 3).

### 3.3. Preliminary Analysis of the Structure of Bacterial Metabolites

The TLC analysis results allow us to make the assumption that *P. chlororaphis* BZR 245-F and *Pseudomonas* sp. BZR 523-2 produce phenazine metabolites. This can be seen when compared with the chromatographic mobility and ultraviolet behavior of standard phenazine. However, there is a need to confirm the results obtained using HPLC-MS.

The diagnostic feature of *Pseudomonas* bacteria is the formation of pigments. The colored substances synthesized by *Pseudomonas* also include vitamins. The composition of the yellow-orange, fluorescent pigment synthesized by *Pseudomonas* includes riboflavin, folic acid, and pterin. Notably, the pigment color may vary depending on the composition of the culture medium. We found a large amount of a bright orange, fluorescent pigment in *P. chlororaphis* BZR 245-F (Rf 0.74). In addition, under UV—366 nm, an intense zone with a dark blue absorption of Rf 0.81 is observed. The remaining fluorescent zones are less pronounced, although there are many of them. An orange, fluorescent pigment was not detected on TLC of the *Pseudomonas* sp. BZR 523-2 supernatant. A low-intensity zone of yellow luminescence (Rf 0.23), also visible on TLC of the *P. chlororaphis* BZR 245-F bacteria supernatant, was observed. Phenazines are simple redox heterocyclic brightly colored pigmented nitrogen-containing compounds with an antibiotic activity [22,23]. The intense zone with dark blue absorption (Rf 0.90) corresponded to that the phenazine standard solution (Merck, Darmstadt, Germany). The remaining zones had a blue glow; the bright blue zone with Rf 0.53 was compact and highly concentrated (Figure 2).

In this regard, it can be assumed that *Pseudomonas* sp. BZR 523-2 produces compounds containing groups of a phenolic nature. The chromatogram of the supernatant of *P. chlororaphis* BZR 245-F bacteria showed a zone that glows in UV366 that is similar to standard phenazine but with a lower chromatographic mobility (Rf 0.85). This is most likely a phenazine compound. Chromatogram analysis under the UV- 254 nm revealed zones of intense absorption in both bacterial strains, which corresponds to the standard phenazine solution in Rf. However, this uptake was much less intense in *Pseudomonas* sp. BZR 523-2 than in *P. chlororaphis* BZR 245-F (Figure 3).

### 3.4. Evaluation of the Antifungal Activity of Bacterial Metabolites by Bioautography

**Figure 4:**
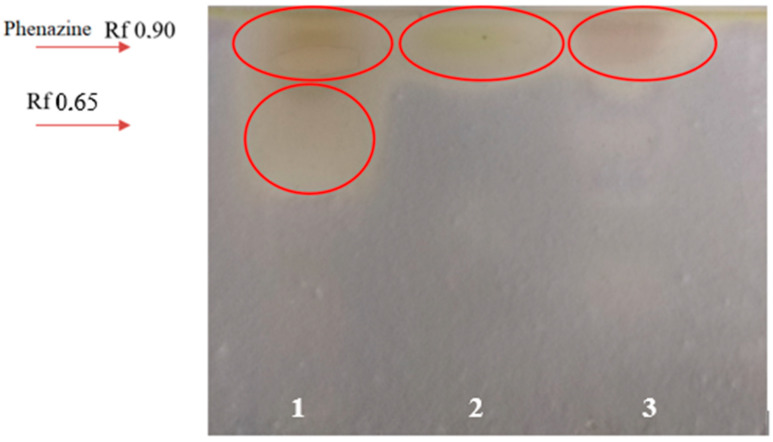
Bioautographs of the ethyl acetate extract of the supernatants of *Pseudomonas* bacteria with a test culture of the *F. oxysporum* var. *orthoceras* BZR F-6 fungus. 1—*P. chlororaphis* BZR 245-F; 2—phenazine standard solution; 3—*Pseudomonas* sp. BZR 523-2. Red circles indicate metabolite zones.

Extensive fungicidal and fungistatic zones were present on the bioautographs of the studied strains (Figure 4). The standard solution of phenazine showed fungicidal properties. For the *P. chlororaphis* BZR 245-F strain, the zone of fungal inhibition of a large area corresponded to the zone of the bright orange pigment on TLC (Rf 0.33–0.68). There were no fungistatic zones for this strain. The *Pseudomonas* sp. BZR 523-2 strain showed a fungicidal zone with an Rf of 0.9. However, this zone had a much smaller area than for *P. chlororaphis* BZR 245-F. This strain is characterized by the presence of insignificant fungistatic zones in relation to the *F. oxysporum* var. *orthoceras* BZR F-6.

Therefore, a preliminary analysis of antifungal metabolites of *P. chlororaphis* BZR 245-F and *Pseudomonas* sp. BZR 523-2 by thin layer chromatography and bioautography reveals significant metabolite activity, especially in *P. chlororaphis* BZR 245-F.

### 3.5. HPLC-MS Analysis of Metabolites of Strain Samples

**Figure 5:**
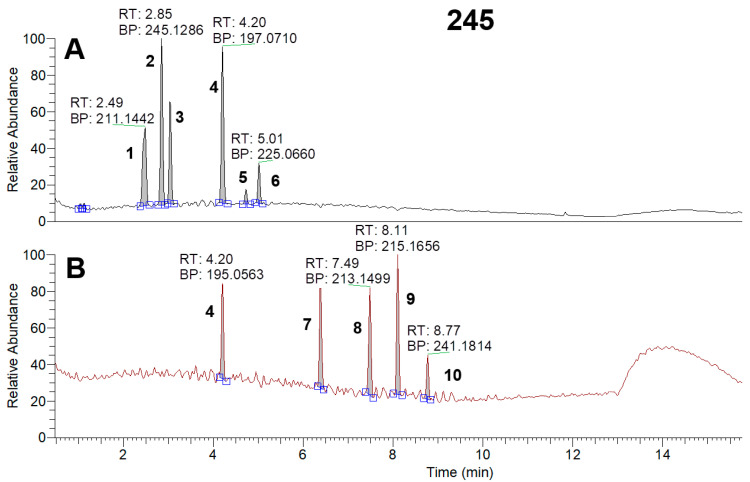
Total ion current chromatograms (100–1000 *m*/*z*) of BZR 245-F sample in positive (**A**) and negative (**B**) ionization modes: 1—Cyclo(Leu-Pro); 2, 3—Cyclo(Phe-Pro); 4—1-Phenazinol; 5—2-Hydroxyphenazine-1-carboxylic acid; 6—Phenazine-1-carboxylic acid; 7—3-Hydroxydecanoic acid; 8—3-Oxododecanoic acid; 9—3-Hydroxydodecanoic acid; 10—3-Oxotetradecanoic acid.

**Figure 6:**
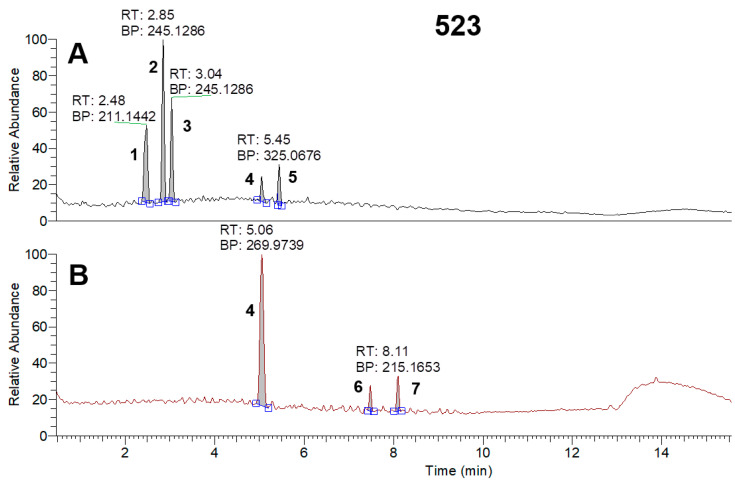
Total ion current chromatograms (100–1000 *m*/*z*) of BZR 523-2 sample in positive (**A**) and negative (**B**) ionization modes: 1—Cyclo(Leu-Pro); 2, 3—Cyclo(Phe-Pro); 4—Pyolyuteorin; 5—Pyocyanin; 6—3-Oxododecanoic acid; 7—3-Hydroxydodecanoic Acid.

**Figure 7:**
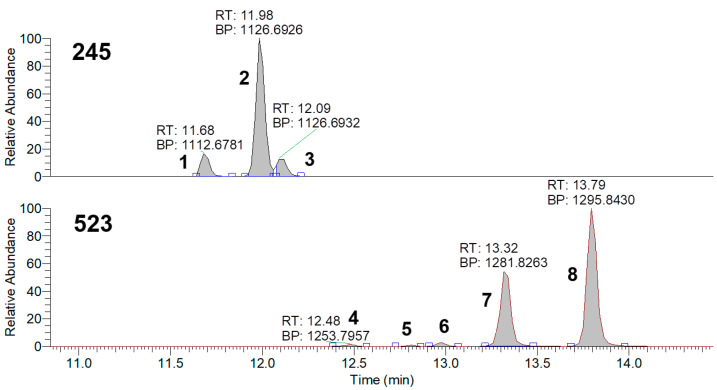
Total ion current chromatograms (400–2000 *m*/*z*) of BZR 245-F and BZR 523-2 samples in positive ionization mode: 1—Massetolide E; 2, 3—Massetolide F or Viscose; 4—Orfamide D; 5—Orfamide E; 6—Orfamide C; 7—Orfamide B; 8—Orfamide A.

*Pseudomonas* strains are capable of producing a large number of bioactive compounds from relatively small molecules, such as phenazines (180.2 g/mol), to relatively big ones, such as viscosin lipopeptides (1126.4 g/mol) [24,25]. Based on the large difference in the masses of the metabolites, for better method sensitivity, it was decided to analyze the samples in two MS conditions: full MS 100–1000 and 400–2000 *m*/*z* followed by DDA. HPLC-MS identification of the metabolites isolated from the supernatants revealed the presence of phenazine and other compounds with similar structures (pyocyanin, phenazine-1-carboxylic acid, etc.), cyclic dipeptides (cyclo(Phe-Pro), cyclo (Pro-Tyr) etc.), pyoluteorin, and lipopeptides (viscosins, massetolides, etc.) (Figure 5, Figure 6 and Figure 7, Table S1).

We found crucial differences in the variety of metabolites in the phenazine structures. In the supernatants of the *P. chlororaphis* BZR 245-F strain, antifungal compounds of a phenazine nature, such as phenazine, pyocyanin, phenazine-1-carboxylic acid, 1-phenazinol, hydroxyphenazine-1-carboxylic acid and phenazine-1-carboxamide, were detected, whereas in the *Pseudomonas* sp. BZR 523-2, we found only phenazine and pyocyanin (Figures S1–S8). For quantitative analysis of the phenazine metabolites, a calibration curve was constructed against the concentration of the phenazine standard solutions (Figure S9). In the measured interval, the dependence of the signal area with *m*/*z* 181.0766 (corresponding to phenazine) on the concentration is linear with a correlation coefficient R^2^ > 0.99.

The phenazine content in *P. chlororaphis* BZR 245-F was found to be approximately 40 times lower than in the *Pseudomonas* sp. BZR 523-2 strain (Table 3). However, the total concentration of the phenazine structures in terms of phenazine in the *P. chlororaphis* BZR 245-F sample was more than five times greater than in the BZR 523-2.

We also found that the BZR 523-2 strain, as opposed to BZR 245-F, produced pyoluteorin and amino pyrrolnitrin, which inhibit the growth of fungi, bacteria, and nematodes [26]. Both bacterial strains produce dipeptides in approximately the same quantities. We also found that the *P. chlororaphis* BZR 245-F strain produced antifungal lipopeptides, such as massetolides (E, F) and viscosins, as opposed to orphamids (A, B, C, D, E) in *Pseudomonas* sp. BZR 523-2 supernatants (Figure 7 and Figures S10–S17). Fatty acids were observed as well, their amount being higher for the *P. chlororaphis* BZR 245-F strain. We assume that the detected fatty acids might be either individual molecules or decomposition products of rhamnolipids [27,28]. All the metabolites that were found during these experiments are presented in Table S1.

## 4. Discussion

Biocontrol strains are typically more effective when the beneficial microbiota simultaneously possess several biocontrol mechanisms or have a wide range of metabolites produced. The approach that we used in this work is that, after isolating metabolites of the biocontrol agents by TLC, we preliminarily analyzed their antifungal activity by bioautography. It speeds up the selection of active strains. Using a pathogen of a harmful plant disease as a test culture for bioautography can immediately provide an answer to the question of the potential effectiveness of the studied microorganisms. These methods allowed for detecting the antifungal activity of the metabolite profiles of the *P. chlororaphis* BZR 245-F and *Pseudomonas* sp. BZR 523-2 bacteria during preliminary analysis. The *P. chlororaphis* BZR 245-F and *Pseudomonas* sp. BZR 523-2 bacterial strains studied produce compounds of phenazine structures, especially abundant for *P. chlororaphis* BZR 245-F. These are redox nitrogen-containing heterocyclic molecules that are capable of suppressing a wide variety of plant pathogens (bacterial, fungal, and oomycete) due to their ability to generate reactive oxygen species [29].

The nature of several chemical functional groups is likely to influence the chemical properties of various phenazine compounds, such as redox activity, solubility, and the ability to move across biological membranes. Phenazines also significantly contribute to the ‘lifestyle’ of the bacteria that produce them. Thus, phenazines promote the formation and growth of biofilms in anoxic conditions. They also play an important role in the stability of the rhizosphere and the release of extracellular DNA. Phenazine production also transforms the expression of many genes, including those involved in the response to oxidative stress, cell autolysis, the production of other secondary metabolites, and iron transportation and efflux mechanisms [10,30,31].

Different research studies show that phenazines exhibit a broad-spectrum antibiotic activity against multiple fungal, bacterial, and oomycete plant pathogens, including *Gaeumannomyces graminis* var. *tritici*, *Rhizoctonia solani*, *Fusarium oxysporum* f. sp. *radicis*—*lycopersici*, *Pythium* spp., and *Phytophthora infestans* [32,33,34,35,36]. The ability of bacteria strains *P. chlororaphis* BZR 245-F and *Pseudomonas* sp. BZR 523-2 to inhibit the growth of the phytopathogenic fungus *F. oxysporum* var. *orthoceras* (Figure 4, Table 3), which is probably provided by phenazine metabolites, allows us to classify them as promising agents for the biocontrol of fungal diseases.

The bacteria strains *P. chlororaphis* BZR 245-F and *Pseudomonas* sp. BZR 523-2 also produce significant amounts of antifungal lipopeptides. This is confirmed by the HPLC and bioautography of metabolites isolated by TLC. At the same time, the structure of these substances is quite diverse.

Most strains of *Pseudomonas* are reported to produce a single cyclic lipopeptide belonging to the groups of viscosins (massetolides) (*P. lactis* SS101), orfamides (*P. protegens* Pf-5 and CHA0), putisolvins (*P. putida* WCU_64), and xantholysins (*P. mossselii* BW11M1) [35]. HPLC combined with nuclear magnetic resonance and time-of-flight mass spectrometry identified an insecticidal biosurfactant produced by *P. protegens* F6 [37]. Metabolite profiling of *P. chlororaphis* BZR 245-F and *Pseudomonas* sp. BZR 523-2 suggests that they can simultaneously produce metabolites that are members of different families of lipopeptides, as well as multiple structural analogs of one particular lipopeptide. This may contribute to higher efficiency and inhibition of a wider range of phytopathogens. An important fact is the presence of fatty acids in the liquid culture of both *P. chlororaphis* BZR 245-F and *Pseudomonas* sp. BZR 523-2. They were identified by HPLC-MS (Supplementary Table S1) and are likely precursors for lipopeptide synthesis.

Thus, metabolite profiling of *P. chlororaphis* BZR 245-F and *Pseudomonas* sp. BZR 523-2 shows that both strains produce a set of chemically diverse bioactive metabolites. This, in turn, demonstrates their ability to protect agricultural crops against phytopathogenic microorganisms both directly and indirectly. Therefore, *P. chlororaphis* BZR 245-F and *Pseudomonas* sp. BZR 523-2 bacteria can be used as producers of effective biopesticides for sustainable agriculture management. As such, the reported results call for further research on these promising biocontrol agents in laboratory and field experiments.

## Figures and Tables

**Table 1:** Antagonistic activity (growth inhibition of two indigenous *Pseudomonas* strains) in dual cultures against *Fusarium* fungi.

Antagonistic Bacterial Strain	Antagonistic Activity against *F. graminearum* BZR F-4, %				Antagonistic Activity against *F. oxysporum* var. *orthoceras* BZR F-6, %			
	5 Days		10 Days		5 Days		10 Days	
	Growth of Fungal Strain	% Inhibition	Growth of Fungal Strain	% Inhibition	Growth of Fungal Strain	% Inhibition	Growth of Fungal Strain	% Inhibition
Negative control (without bacterial strains)	50.0 ± 0.6 a	–	75.0 ± 0 a	–	46.7 ± 3.0 a	–	75.0 ± 0 a	–
*P. chlororaphis* BZR 245-F	38.7 ± 3.5 b	22.7	30.3 ± 1.5 c	59.6	33.7 ± 1.5 b	27.9	37.3 ± 4.7 c	50.2
*Pseudomonas* sp. BZR 523-2	43.7 ± 1.2 b	12.7	63.7 ± 2.9 b	15.1	35.7 ± 1.2 b	23.6	68.3 ± 0.6 b	8.9

Data followed by the same letters are not statistically different according to Duncan’s multiple range test (*p* = 0.05).

**Table 2:** Effect of bacterial strains on growth and dry biomass of winter wheat plants in the pot experiment.

Treatment	Plant Height, mm	Root Length, mm	Weight of Dry Biomass, g	
			Shoots	Roots
Control	23.3 ± 0.3 a	12.9 ± 0.6 a	0.0161 ± 0.002 a	0.0083 ± 0.0005 a
*P. chlororaphis* BZR 245-F	24.1 ± 0.2 b	11.9 ± 0.4 a	0.0166 ± 0.002 a	0.0089 ± 0.0002 a
*Pseudomonas* sp. BZR 523-2	25.7 ± 0.5 c	15.3 ± 0.5 b	0.0197 ± 0.003 b	0.0094 ± 0.0001 b

Data followed by the same letters are not statistically different according to Duncan’s multiple range test (*p* = 0.05).

**Table 3:** Content of phenazine nature metabolites in mg to g of dry residues of *P. chlororaphis* BZR 245-F and *Pseudomonas* sp. BZR 523-2 determined by HPLC-MS.

Compound	Structure and Gross Formula	Content, mg/g	
		*P. chlororaphis* BZR 245-F	*Pseudomonas* sp. BZR 523-2
Phenazine	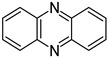 C_12_H_8_N_2_	0.07	2.77
Pyocyanin	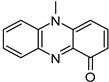 C_13_H_10_N_2_O	0.16	0.14
Phenazine-1-carboxylic acid	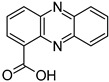 C_13_H_8_N_2_O_2_	3.22	–
1-Phenazinol	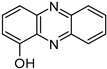 C_12_H_8_N_2_O	11.3	–
2-Hydroxyphenazine-1-carboxylic acid	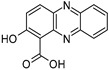 C_13_H_8_N_2_O_3_	2.26	–
Phenazine-1-carboxamide	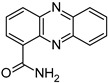 C_13_H_9_N_3_O	0.01	–

## Data Availability

Datasets analyzed or generated during the study did not create on links to publicly archived.

## Supplementary Materials

The following supporting information can be downloaded at: https://www.mdpi.com/article/10.3390/microorganisms11081943/s1, Table S1, Figures S1–S8, Figures S10–S17: HPLC-MS analysis of bacterial metabolites of *P. chlororaphis* BZR 245-F and *Pseudomonas* sp. BZR 523-2, Figure S9: Dependency graph of the signal area with *m*/*z* 181.0766 on the concentration of standard phenazine.
