# Supplementary material for: New Pseudomonas Bacterial Strains: Biological Activity and Characteristic Properties of Metabolites
Source: Microorganisms. 2023 Jul 29;11(8):1943. doi: 10.3390/microorganisms11081943 (PMC10459626; doi:10.3390/microorganisms11081943)
Supplement: Supplementary file 1 [file microorganisms-11-01943-s001.zip › microorganisms-2489372-supplementary.pdf]

Supplementary Materials to the manuscript

New Pseudomonas Bacterial Strains: Biological Activity and Characteristic Properties of Metabolites

Tatiana M. Sidorova [1], Natalia S. Tomashevich [1,*], Valeria V. Allahverdyan [1], Boris S. Tupertsev [2,3], Yuri I. Kostyukevich [3] and Anzhela M. Asaturova [1]

[1] Federal Research Center of Biological Plant Protection, 350039 Krasnodar, Russia

[2] Phystech School of Biological and Medical Physics (FBMF), Moscow Institute of Physics and Technology, 141701 Dolgoprudny, Russia

[3] Center of Molecular and Cellular Biology (CMCB), Skolkovo Institute of Science and Technology, 121205 Moscow, Russia

* Correspondence: tom-s2@yandex.ru; Tel.: +7-92-8038-8165

Table S1. The results of the analysis of *P. chlororaphis* BZR 245-F and *Pseudomonas* sp. BZR 523-2. C.u , are area conventional units obtained by Q Exactive Orbitrap mass spectrometer

Sample	Assignment, Structure	Molecular Formula			RT, min	Area, c.u., amount
		m/z_{ob} (Ionization Mode)	m/z_{th}	Error (ppm)		
245	Phenazine 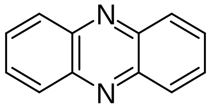	$C_{12}H_8N_2$			5.07	2396173<br>0.07 mg/g
		181.0760 (+)	181.0760	0		
	Pyocyanin 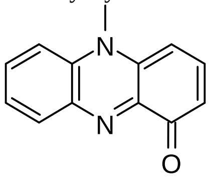	$C_{13}H_{10}N_2O$			5.46	5126746<br>0.16 mg/g
		211.0864 (+)	211.0864	0		
	Phenazine-1-carboxylic acid 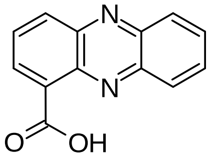	$C_{13}H_8N_2O_2$			5.01	104904177<br>3.22 mg/g
		225.0660 (+)	225.0659	0.4		
	1-Phenazinol 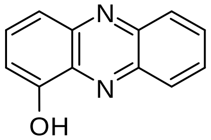	$C_{12}H_8N_2O$			4.20	300012231<br>11.27 mg/g
		197.0709 (+)	197.0709	0		
	2-Hydroxyphenazine-1-carboxylic acid 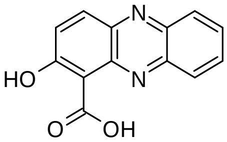	$C_{13}H_8N_2O_3$			4.73	74811412<br>2.26 mg/g
		241.0609 (+)	241.0608	0.4		
	Phenazine-1-carboxamide 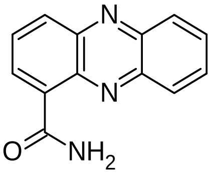	$C_{13}H_9N_3O$			4.52	282099<br>14.8 mg/g
		224.0816 (+)	224.0818	-0.9		
	Cyclo (Leu-Pro) 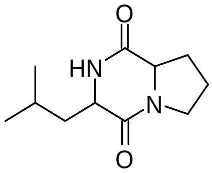	$C_{11}H_{18}N_2O_2$			2.49	271658961
		211.1441 (+)	211.1441	0		
	Cyclo (Leu-4-OH-Pro) 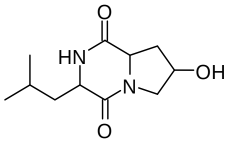	$C_{11}H_{18}N_2O_3$			1.43	24334107
		227.1389 (+)	227.1390	-0.4		
	Cyclo (Pro-Val)	$C_{10}H_{16}N_2O_2$			1.22	18127820

	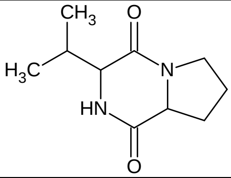	197.1283 (+)	197.1285	-1.0		
	Cyclo (Phe-Pro) 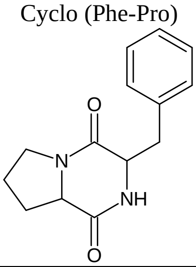	$C_{14}H_{16}N_2O_2$			2.85<br>3.04	297513831<br>199652217<br>Sum 499190896
		245.1285 (+)	245.1285	0		
	Cyclo (Tyr-Pro) 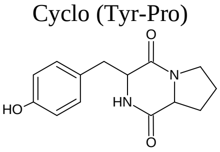	$C_{14}H_{16}N_2O_3$			1.06<br>1.11<br>1.92<br>2.13	25762123<br>28104244<br>4903739<br>14954527<br>Sum 73724633
		261.1234 (+)	261.1234	0		
	Massetolide F/Viscosin 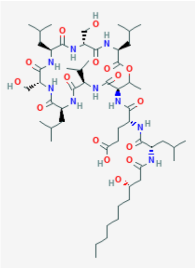	$C_{54}H_{95}N_9O_{16}$			11.98	2287283
		1126.6975 (+)	1126.6970	0.4		
	Massetolide F/Viscosin 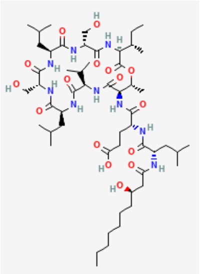	$C_{54}H_{95}N_9O_{16}$			12.09	371552
		1126.6951 (+)	1126.6970	-1.7		
	Massetolide E 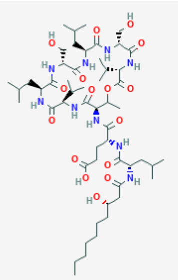	$C_{53}H_{93}N_9O_{16}$			11.68	344023
		1112.6814 (+)	1112.6813	0.1		
	3-Hydroxydecanoic acid 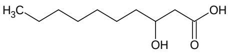	$C_{10}H_{20}O_3$			6.38	24706173
		187.1338 (-)	187.1340	-1.1		

	3-Oxododecanoic acid 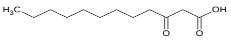	$C_{12}H_{22}O_3$			7.49	23474555
		213.1499 (-)	213.1496	1.4		
	3-Hydroxydodecanoic acid 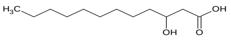	$C_{12}H_{24}O_3$			8.11	29080139
		215.1656 (-)	215.1653	1.4		
	3-Oxotetradecanoic acid 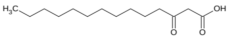	$C_{14}H_{26}O_3$			8.77	13172823
		241.1814 (-)	241.1809	2.1		
523	Phenazine 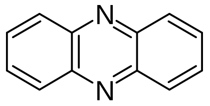	$C_{12}H_8N_2$			5.05	73450415<br>2.77 mg/g
		181.0760	181.0760	0		
	Pyocyanin 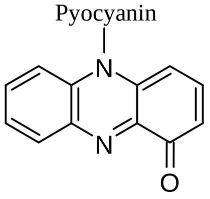	$C_{13}H_{10}N_2O$			5.47	3482940<br>0.14 mg/g
		211.0864 (+)	211.0864	0		
	Cyclo (Leu-Pro) 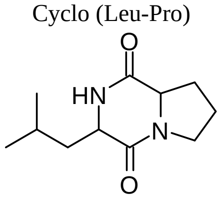	$C_{11}H_{18}N_2O_2$			2.48	343763264
		211.1441 (+)	211.1441	0		
	Cyclo (Leu-4-OH-Pro) 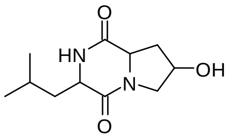	$C_{11}H_{18}N_2O_3$			1.43	36169955
		227.1389 (+)	227.1388	0.4		
	Cyclo (Pro-Val) 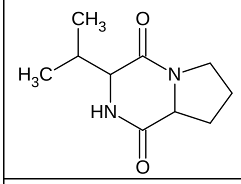	$C_{10}H_{16}N_2O_2$			1.22	23544170
		197.1283	197.1285	-1.0		
	Cyclo (Phe-Pro)	$C_{14}H_{16}N_2O_2$			2.85	374 609 480
		245.1285 (+)	245.1285	0	3.04	246 593 042<br>Sum

	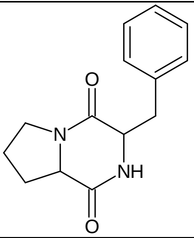					621202523
	Cyclo (Tyr-Pro) 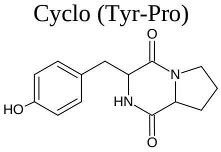	$C_{14}H_{16}N_2O_3$			1.04<br>1.13<br>1.93<br>2.11	34 527 281<br>37 763 732<br>6 023 925<br>23 816 480<br><br>Sum<br>102131419
		261.1234	261.1234	0		
	Piolyuteorin 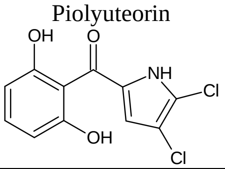	$C_{11}H_7Cl_2NO_3$			5.05	84541796
		271.9876 (+)	271.9876	0		
	3-Chloro-4(2-amino-3-chlorophenyl)-pyrrole 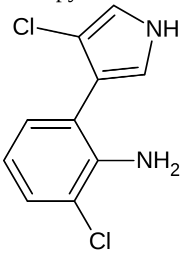	$C_{10}H_8Cl_2N_2$			7.25	3483311
		227.0135 (+)	227.0137	-0.9		
	3-hydroxydecanoic acid 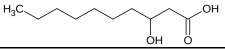	$C_{10}H_{20}O_3$			6.38	5170154
		187.1338 (-)	187.1340	-1.1		
	3-Oxododecanoic acid 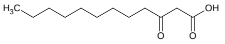	$C_{12}H_{22}O_3$			7.48	15738884
		213.1499 (-)	213.1496	1.4		
	3-Hydroxydodecanoic acid 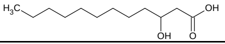	$C_{12}H_{24}O_3$			8.11	20547749
		215.1656 (-)	215.1653	1.4		
	3-Oxotetradecanoic acid 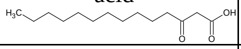	$C_{14}H_{26}O_3$			8.77	6050246
		241.1814 (-)	241.1809	2.1		
	Orfamide A 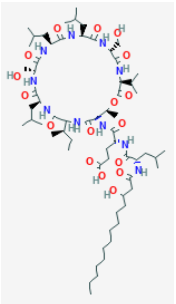	$C_{64}H_{114}N_{10}O_{17}$			13.87	11559958
		1295.8421 (+)	1295.8436	-1.2		
	Orfamide B	$C_{63}H_{112}N_{10}O_{17}$			13.32	11334776
		1281.8271 (+)	1281.8280	-0.7		

	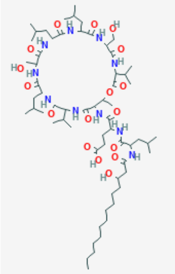					
	Orfamide C 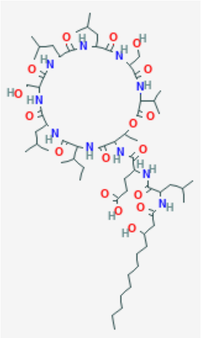	$C_{62}H_{110}N_{10}O_{17}$			13.00	897960
		1267.8113 (+)	1267.8123	-0.8		
	Orfamide D 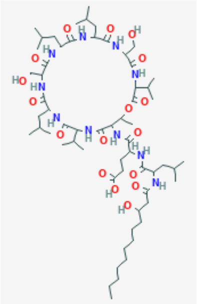	$C_{61}H_{108}N_{10}O_{17}$			12.48	644091
		1253.7960 (+)	1253.7967	-0.6		
	Orfamide E 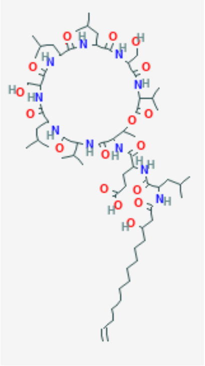	$C_{63}H_{110}N_{10}O_{17}$			12.87	221483
		1279.8116 (+)	1279.8123	-0.5		

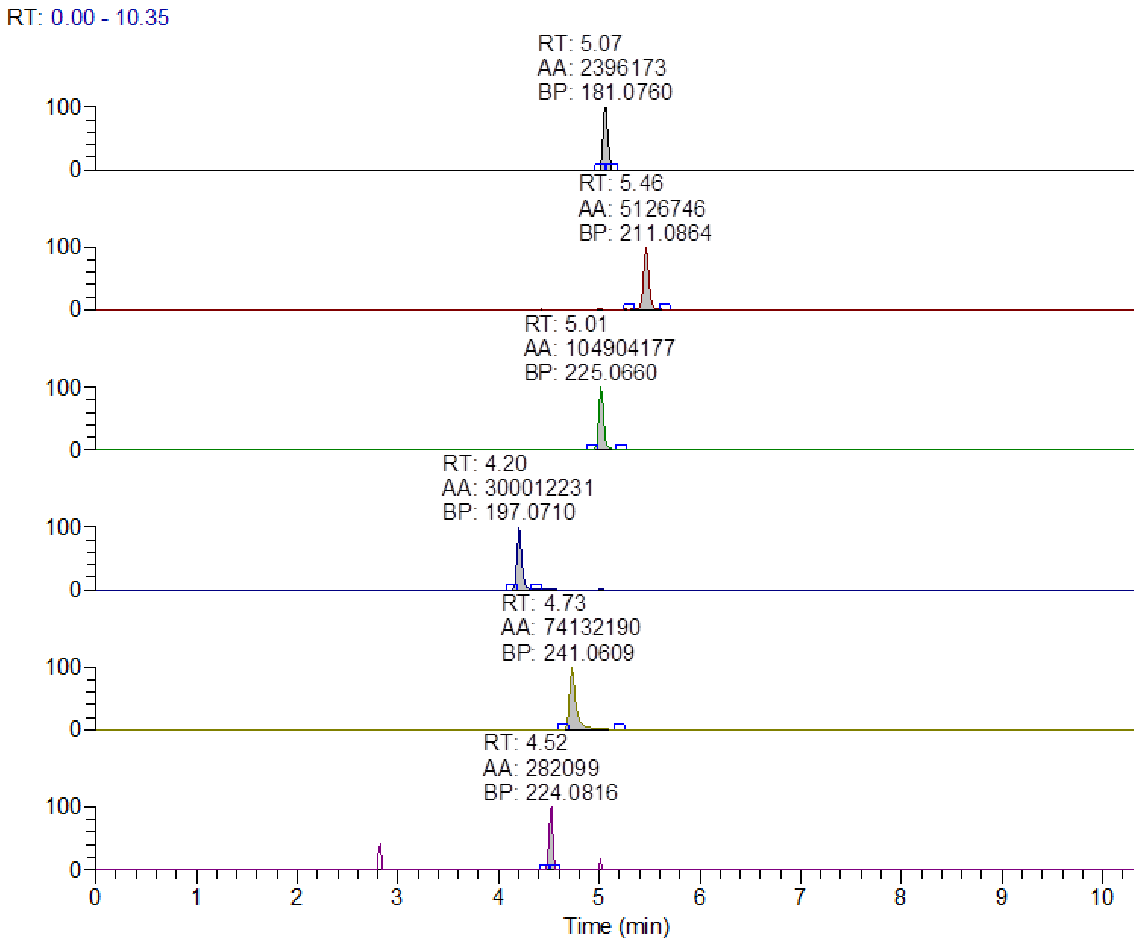

Figure S1 – Base Peak mass chromatograms of *P. chlororaphis* BZR 245-F with m/z 181.0760 (Phenazine), 211.0864 (Pyocyanin), 225.0659 (Phenazine-1-carboxylic acid), 197.0710 (1-Phenazinol), 241.0608 (2-Hydroxyphenazine-1-carboxylic acid), 224.0818 (Phenazine-1-carboxamide)

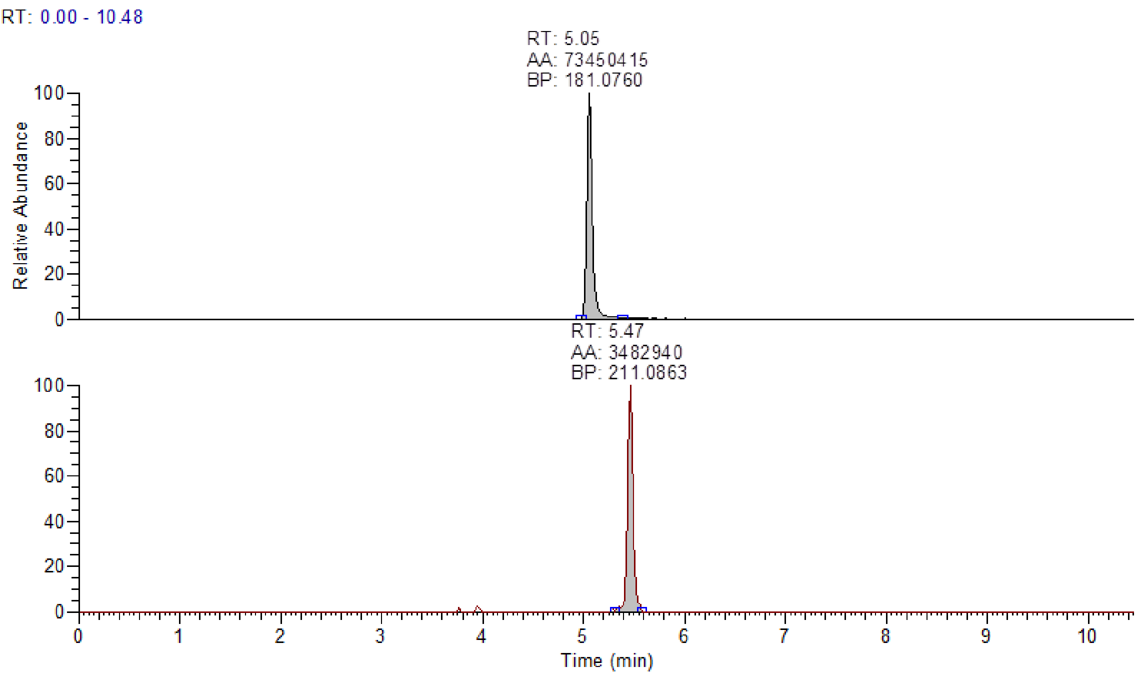

Figure S2 – Base Peak mass chromatograms of *Pseudomonas* sp. BZR 523-2 with m/z 181.0760 (Phenazine), 211.0864 (Pyocyanin)

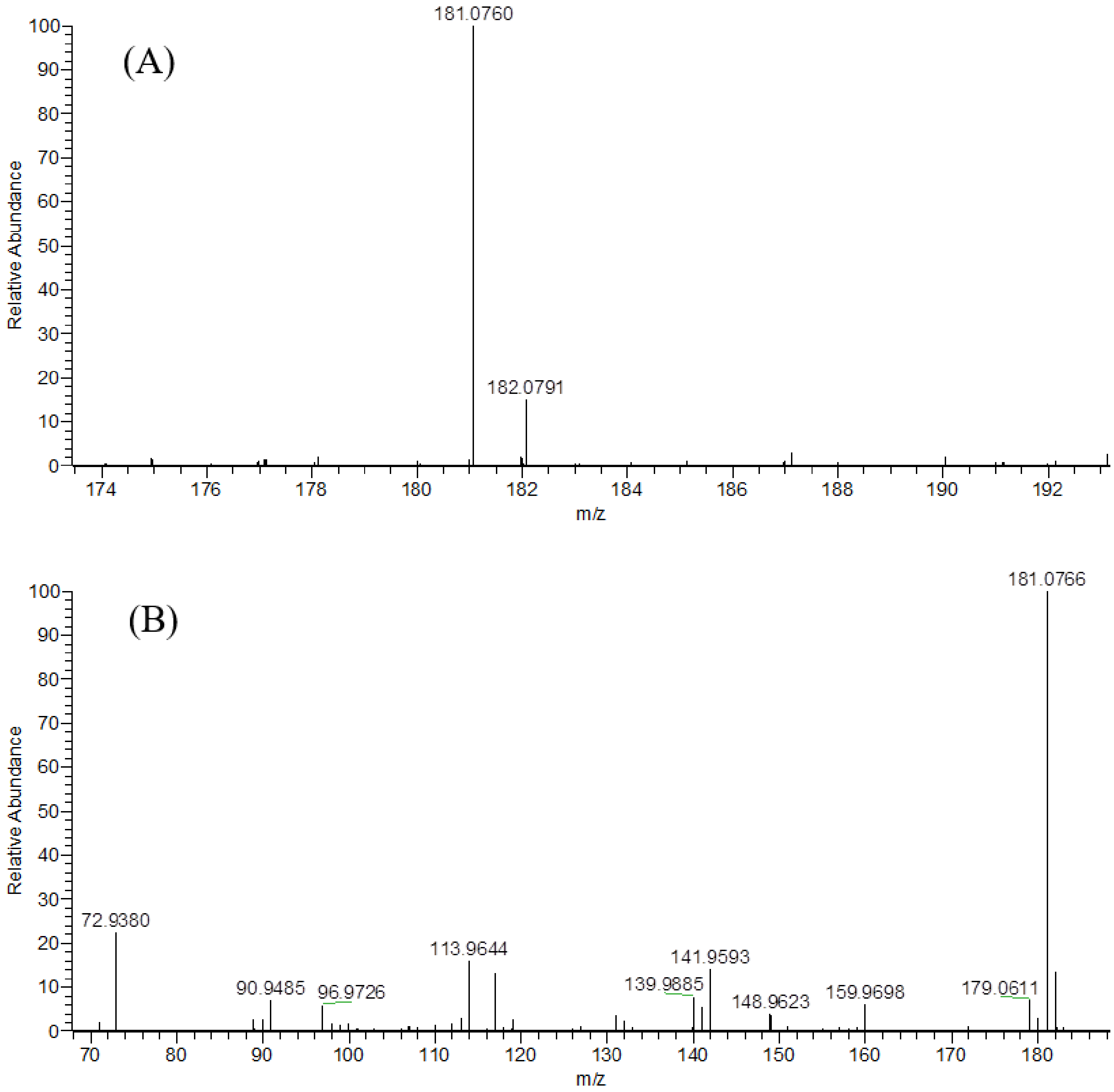

Figure S3 – MS (A) and MS/MS (B) spectra of metabolite with RT 5.05 min from *P. chlororaphis* BZR 245-F. The presented spectra are in positive-ion electrospray ionization mode and equal to those from *Pseudomonas* sp. BZR 523-2 sample

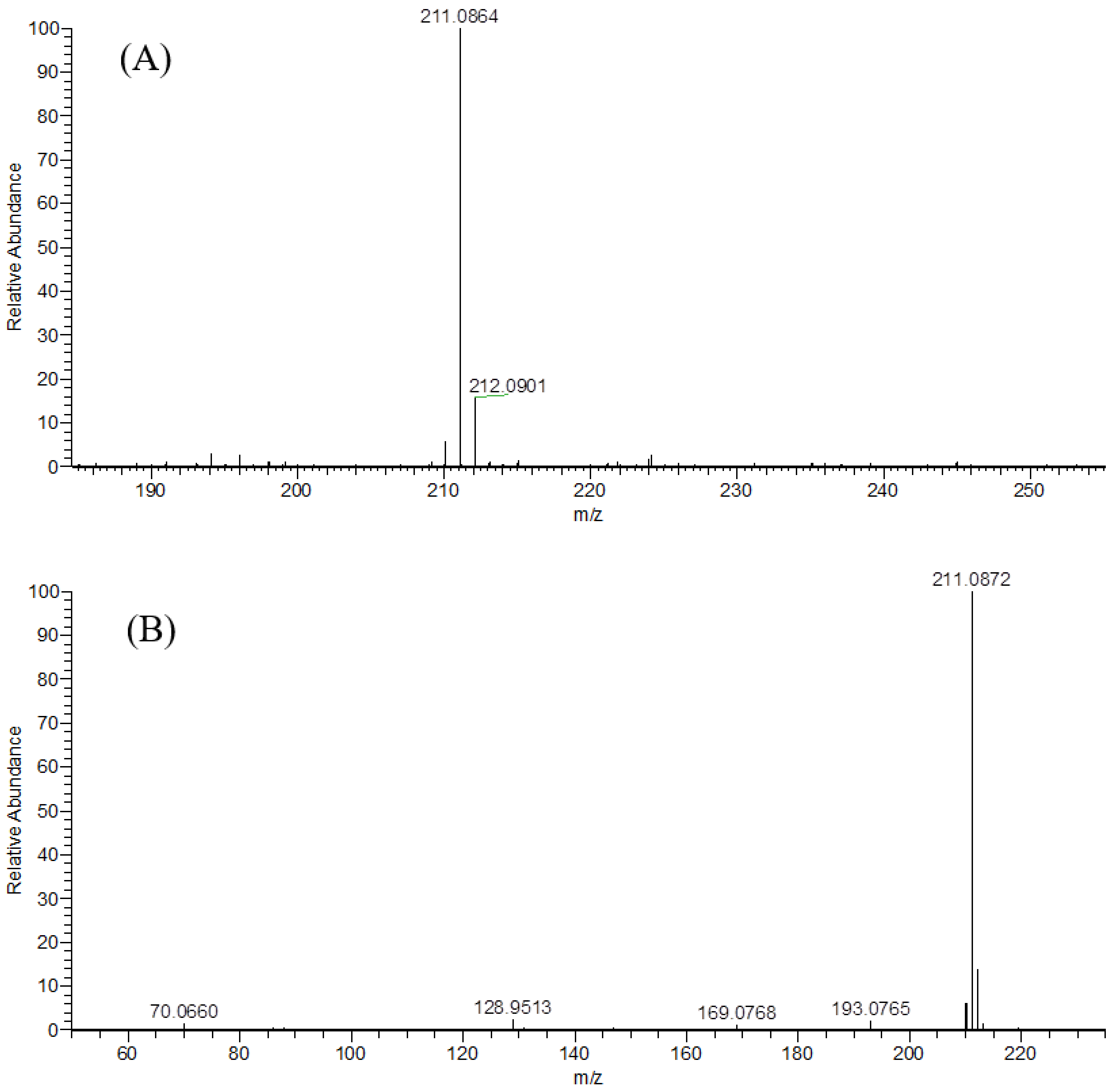

Figure S4 – MS (A) and MS/MS (B) spectra of metabolite with RT 5.45 min from *P. chlororaphis* BZR 245-F. The presented spectra are in positive-ion electrospray ionization mode and equal to those from Pseudomonas sp. BZR 523-2 sample

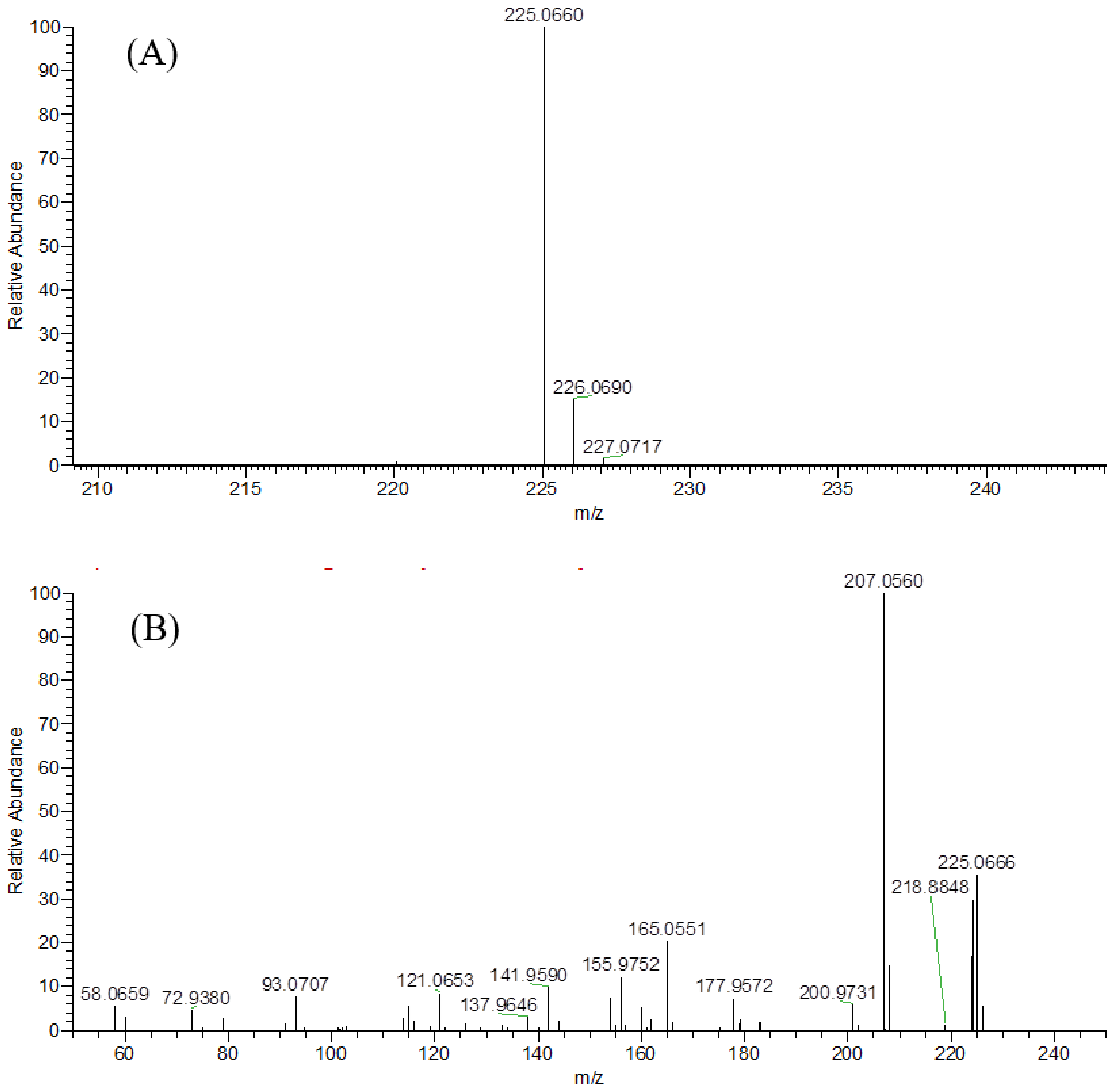

Figure S5 – MS (A) and MS/MS (B) spectra of metabolite with RT 5.01 min from *P. chlororaphis* BZR 245-F. The presented spectra are in positive-ion electrospray ionization mode

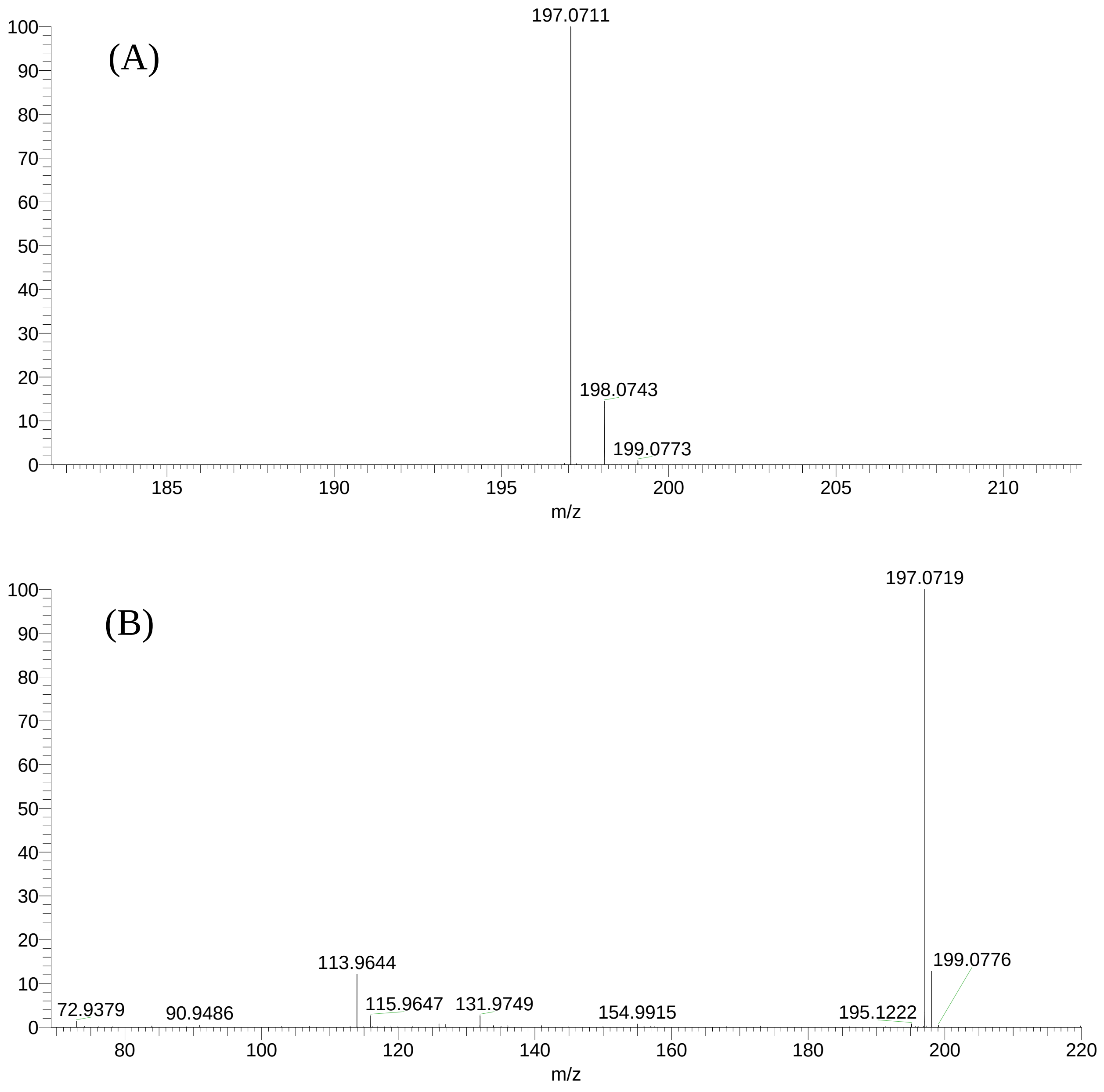

Figure S6 – MS (A) and MS/MS (B) spectra of metabolite with RT 4.20 min from *P. chlororaphis* BZR 245-F. The presented spectra are in positive-ion electrospray ionization mode

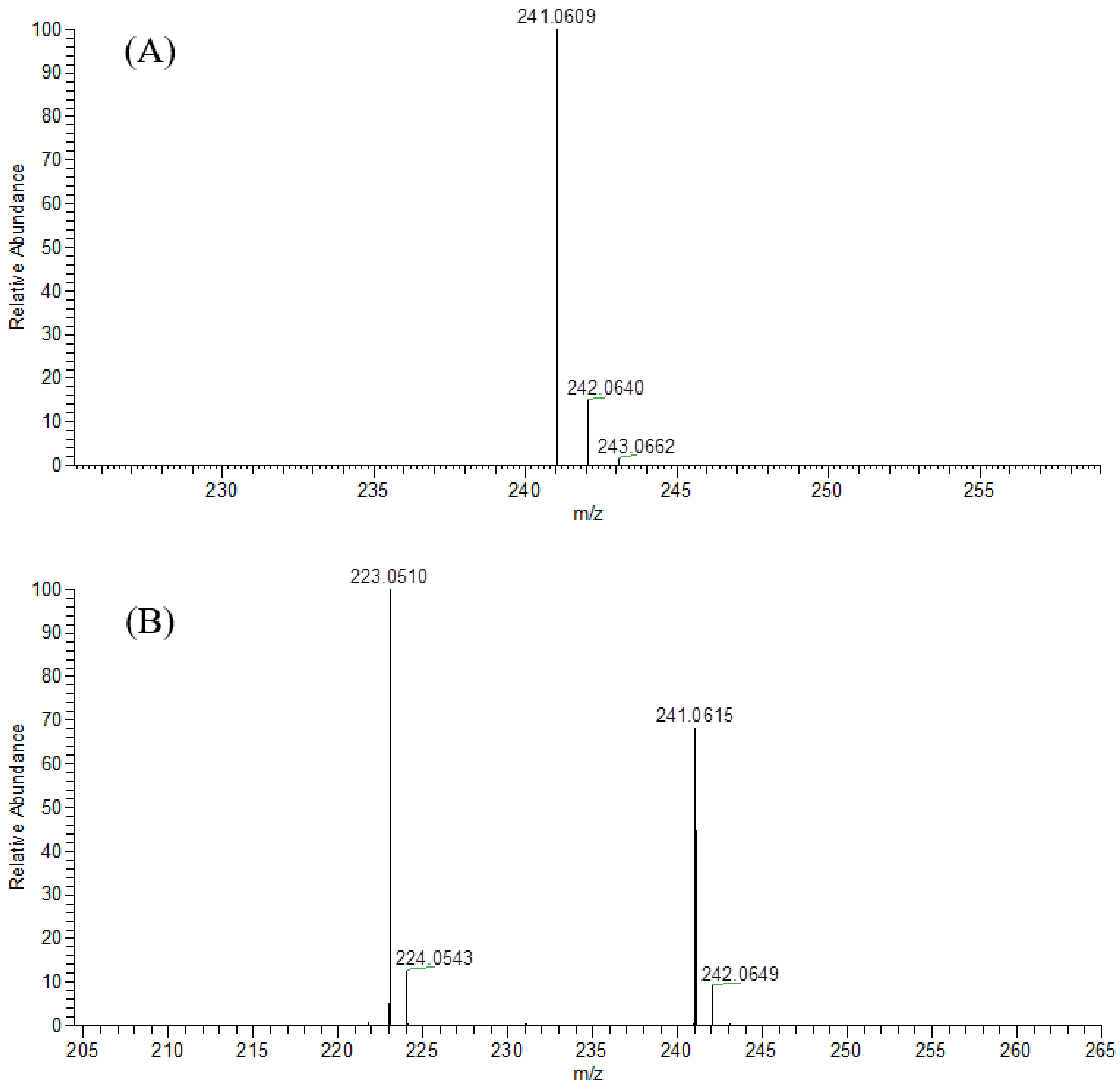

Figure S7 – MS (A) and MS/MS (B) spectra of metabolite with RT 4.73 min from *P. chlororaphis* BZR 245-F. The presented spectra are in positive-ion electrospray ionization mode

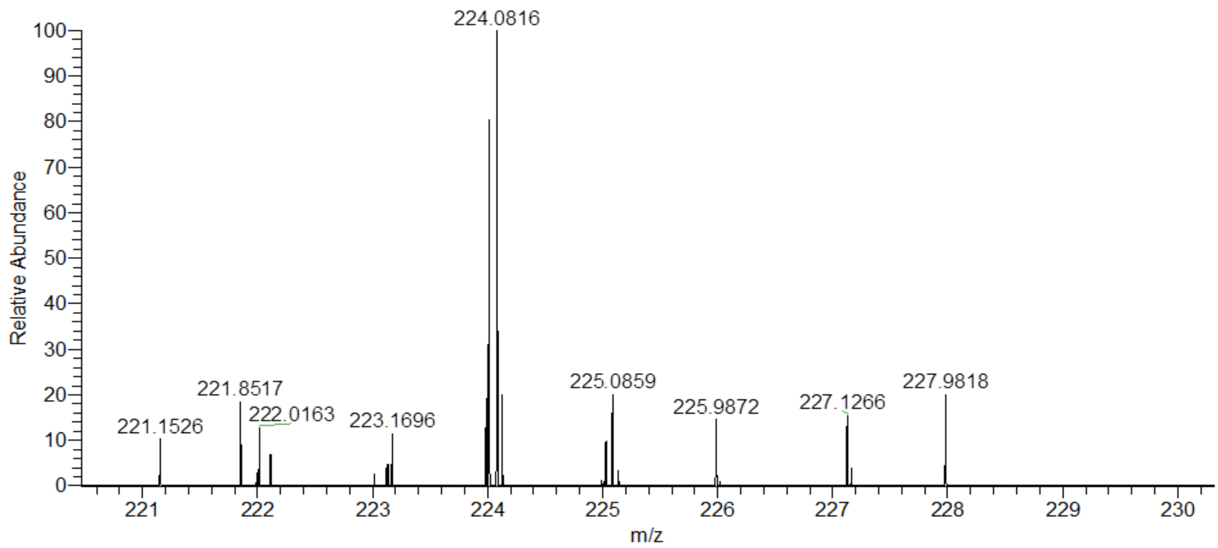

Figure S8 – MS spectrum in positive-ion electrospray ionization mode of metabolite with RT 4.52 min from *P. chlororaphis* BZR 245-F

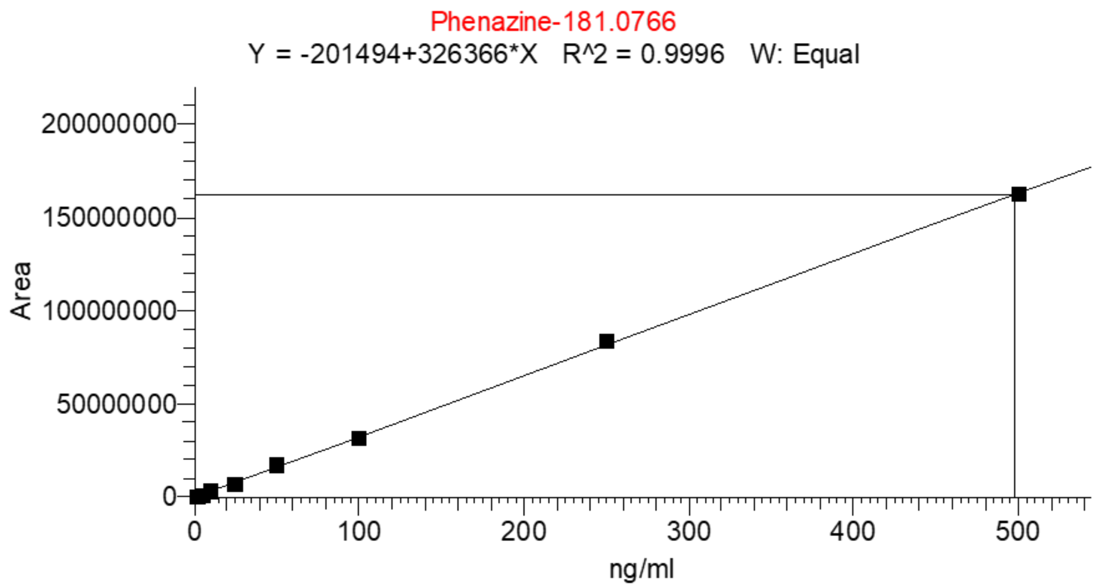

Figure S9 – Dependency graph of the signal area with m / z 181.0766 on the concentration of standard phenazine**.**

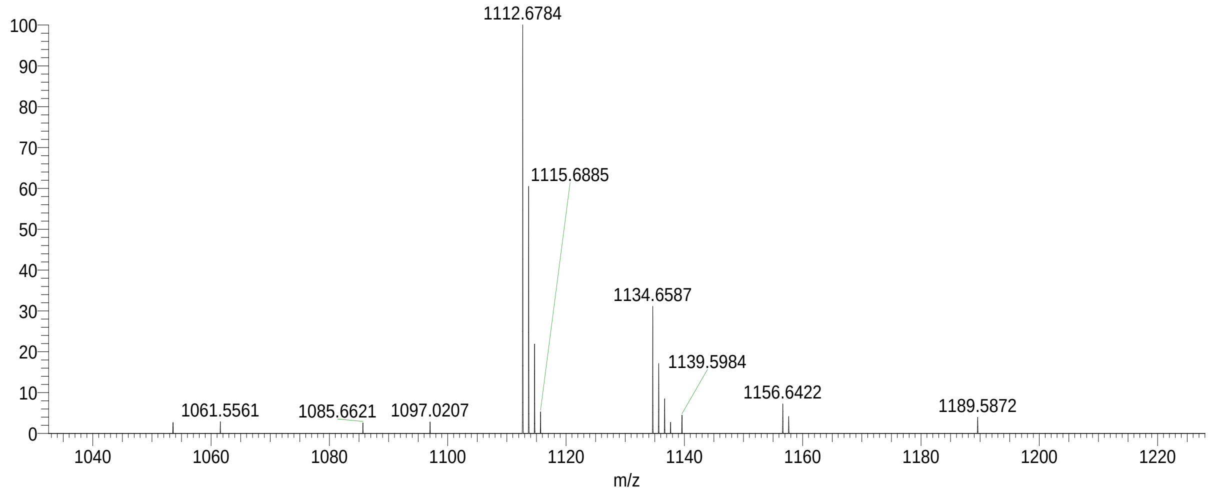

Figure S10 – MS spectrum in positive-ion electrospray ionization mode of metabolite with RT 11.68 min from *P. chlororaphis* BZR 245-F. sample

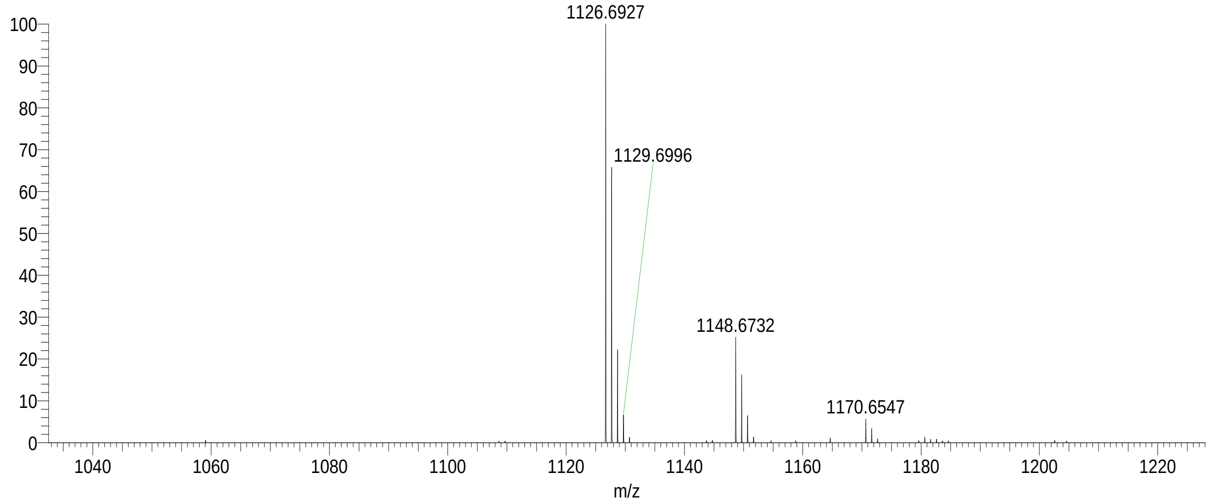

Figure S11 – MS spectrum in positive-ion electrospray ionization mode of metabolite with RT 11.98 min from *P. chlororaphis* BZR 245-F. sample

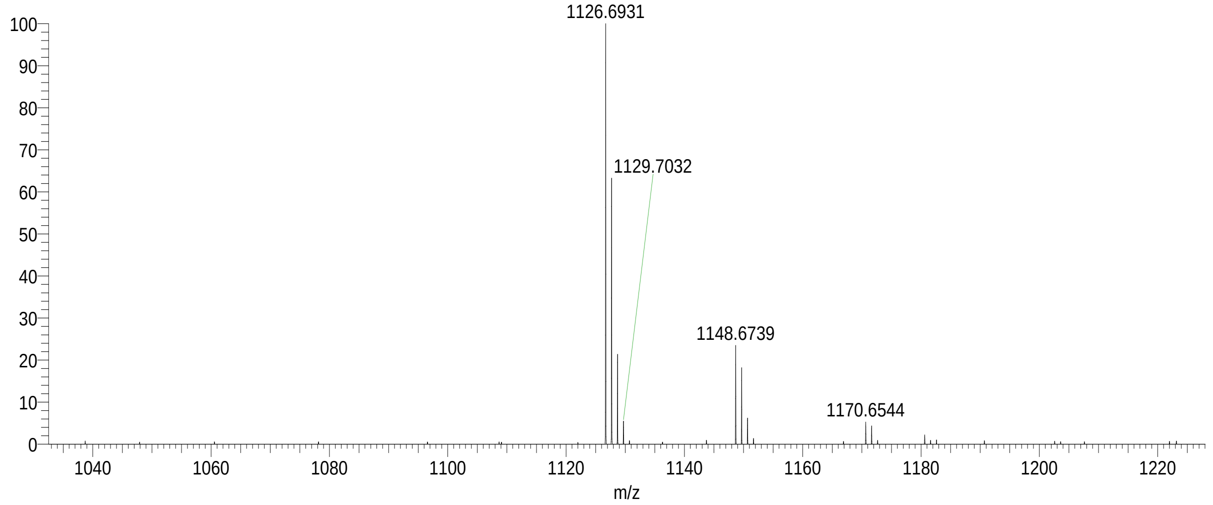

Figure S12 – MS spectrum in positive-ion electrospray ionization mode of metabolite with RT 12.09 min from *P. chlororaphis* BZR 245-F. sample

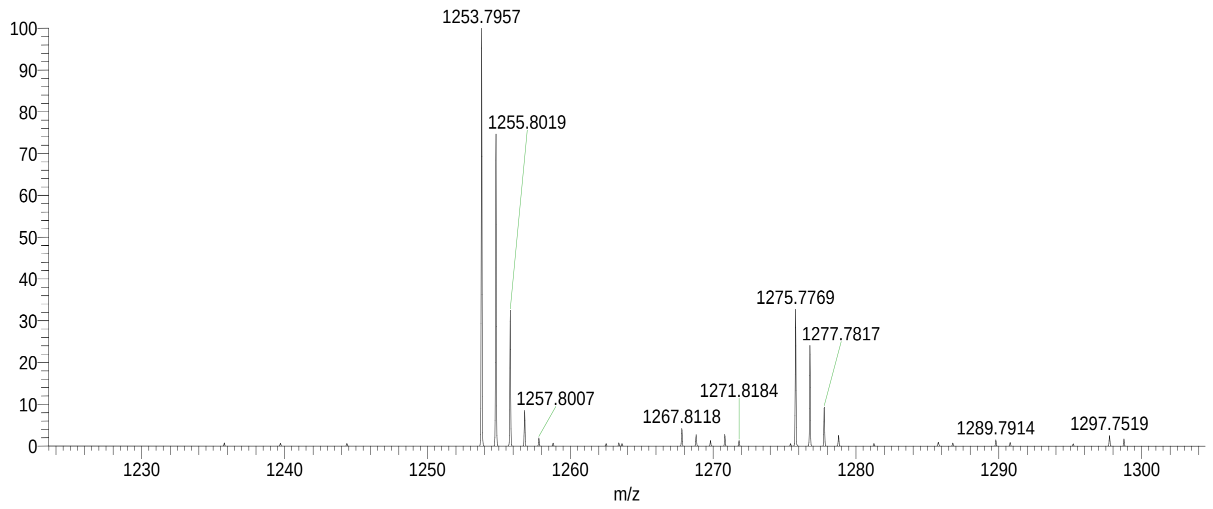

Figure S13 – MS spectrum in positive-ion electrospray ionization mode of metabolite with RT 12.48 min from *Pseudomonas* sp. BZR 523-2 sample

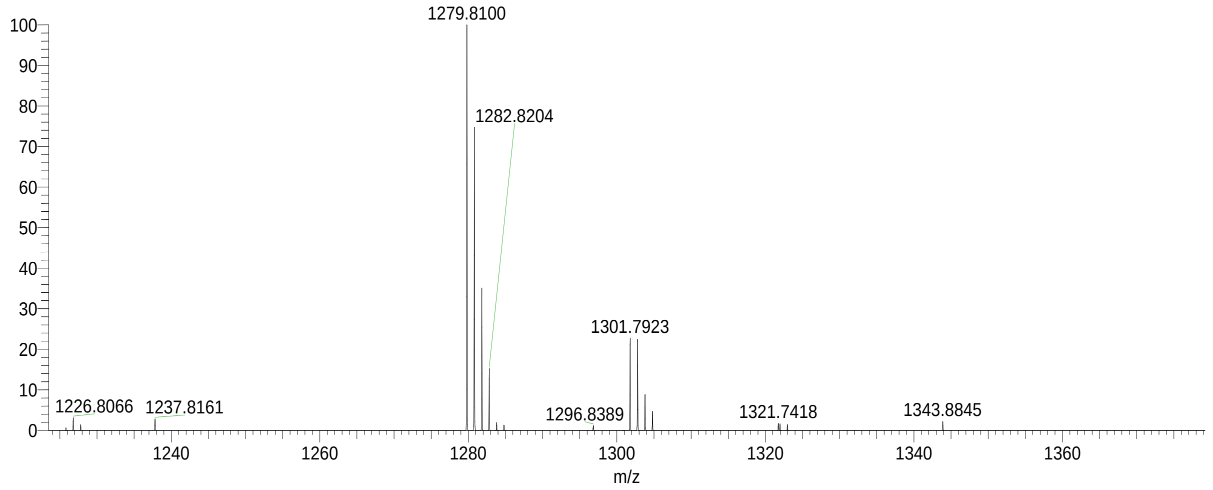

Figure S14 – MS spectrum in positive-ion electrospray ionization mode of metabolite with RT 12.82 min from *Pseudomonas* sp. BZR 523-2 sample

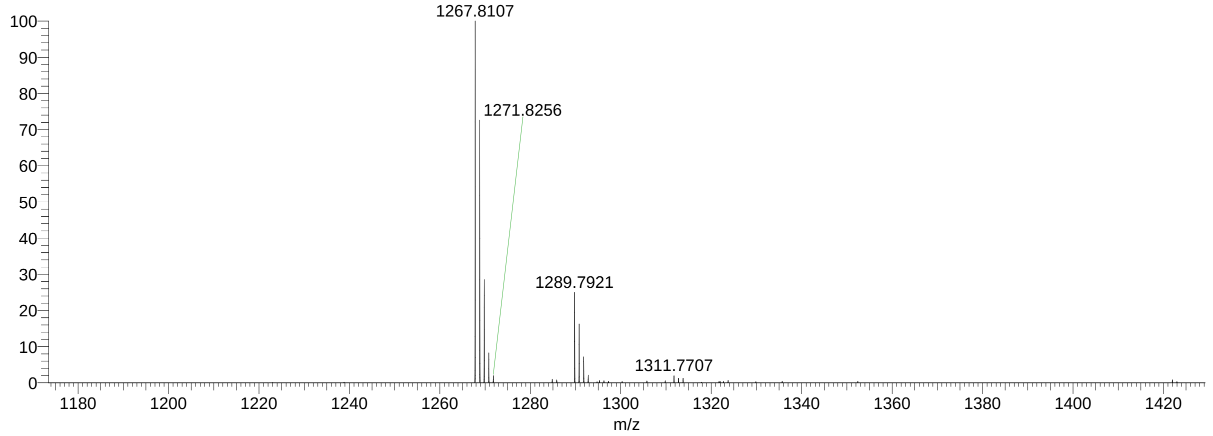

Figure S15 – MS spectrum in positive-ion electrospray ionization mode of metabolite with RT 13.00 min from *Pseudomonas* sp. BZR 523-2 sample

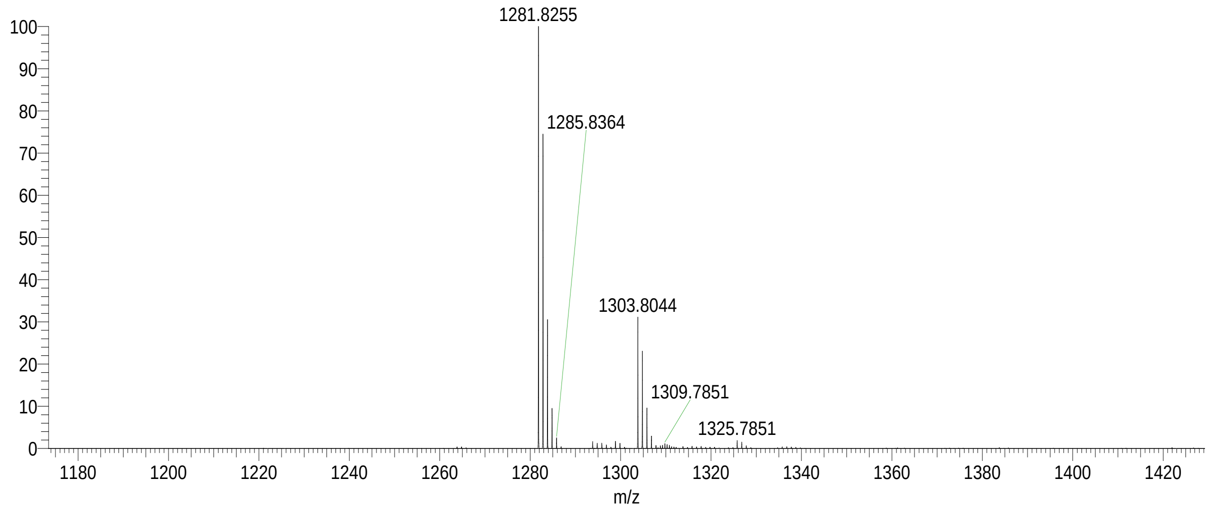

Figure S16 – MS spectrum in positive-ion electrospray ionization mode of metabolite with RT 13.32 min from *Pseudomonas* sp. BZR 523-2 sample

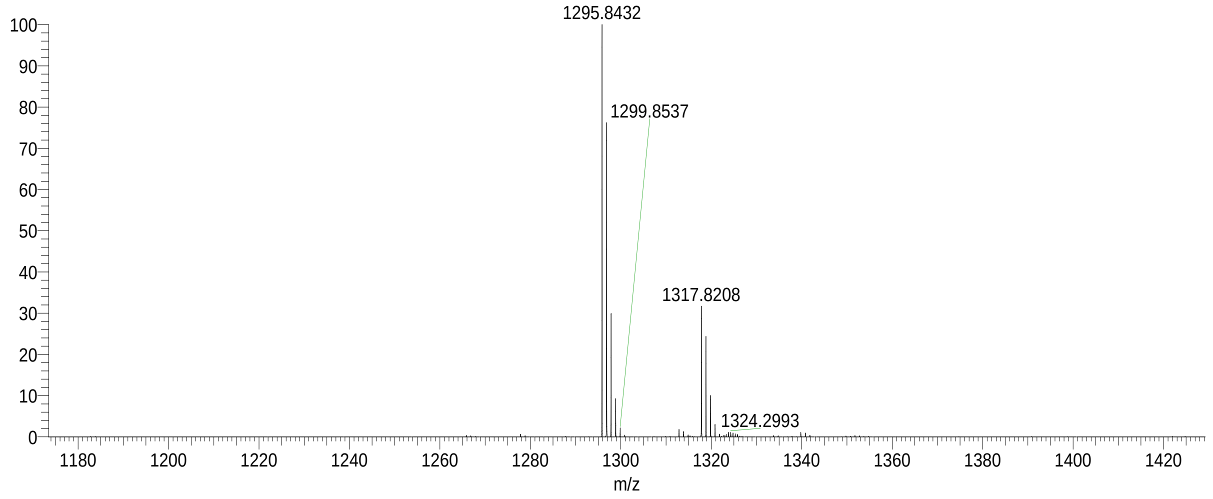

Figure S17 – MS spectrum in positive-ion electrospray ionization mode of metabolite with RT 13.80 min from *Pseudomonas* sp. BZR 523-2 sample
